# Acoustic Classification of Surface and Underwater Vessels in the Ocean Using Supervised Machine Learning

**DOI:** 10.3390/s19163492

**Published:** 2019-08-09

**Authors:** Jongkwon Choi, Youngmin Choo, Keunhwa Lee

**Affiliations:** Department of Defense Systems Engineering, Sejong University, Neungdong-ro 209, Kwangjin-gu, Seoul 05006, Korea

**Keywords:** target depth classification, machine learning, random forest, support vector machine, feed-forward neural network, convolutional neural network, cross-spectral density matrix, vertical line array

## Abstract

Four data-driven methods—random forest (RF), support vector machine (SVM), feed-forward neural network (FNN), and convolutional neural network (CNN)—are applied to discriminate surface and underwater vessels in the ocean using low-frequency acoustic pressure data. Acoustic data are modeled considering a vertical line array by a Monte Carlo simulation using the underwater acoustic propagation model, KRAKEN, in the ocean environment of East Sea in Korea. The raw data are preprocessed and reorganized into the phone-space cross-spectral density matrix (pCSDM) and mode-space cross-spectral density matrix (mCSDM). Two additional matrices are generated using the absolute values of matrix elements in each CSDM. Each of these four matrices is used as input data for supervised machine learning. Binary classification is performed by using RF, SVM, FNN, and CNN, and the obtained results are compared. All machine-learning algorithms show an accuracy of >95% for three types of input data—the pCSDM, mCSDM, and mCSDM with the absolute matrix elements. The CNN is the best in terms of low percent error. In particular, the result using the complex pCSDM is encouraging because these data-driven methods inherently do not require environmental information. This work demonstrates the potential of machine learning to discriminate between surface and underwater vessels in the ocean.

## 1. Introduction

In a shallow-water waveguide, where the boundary interaction is strong, passive sonar usually has difficulty in discriminating a surface source such as a fishery boat or merchant ship from an underwater source such as an unmanned underwater vehicle or submarine. It has been a severe problem for the source detection and tracking using a passive sonar system [1,2,3]. 

A common way to solve this problem is to estimate the depth of a source directly. In underwater acoustics, there exists a traditional method for the source depth estimation called a matched-field processing (MFP), which is a parameter estimation technique that integrates a physical model for acoustic propagation into the signal-processing algorithm [4,5,6,7]. The MFP received a lot of attention due to its high accuracy; however, it suffers from the deterioration caused by the environmental mismatch. As a variant of the MFP, Yang [8] suggests the matched-mode processing (MMP), which uses the normal-mode representation of the acoustic pressure field. Signal processing in the mode-space enhances the signal-to-noise ratio and provides better estimation, which is robust to the environmental mismatch. However, the MMP essentially has the same limitation as the MFP: It requires data of ocean environment such as sound speed, bottom sound speed, bottom density, and water depth, which are used to calculate a physical model. When a *priori* information is biased or incorrect in the MFP or MMP, their results will be unreliable.

Considering that accurate depth estimation is not feasible, it may be a good idea to reformulate the depth estimation problem as a binary classification problem. A few researchers [1,9,10,11,12,13,14,15] have studied the binary classification of surface and underwater sources with model-driven methods. Premus [1] originally introduced a statistical algorithm involving the scintillation index for the source depth discrimination. His algorithm is based on the fact that the time variance of the mode amplitudes for a surface source is larger than for an underwater source. A Monte-Carlo simulation is used to construct an estimated probability density function for a surface or underwater source. In 2007, Premus and Backman [9] presented new depth discrimination approach using the normal-mode subspace model. Generally, a typical shallow-water waveguide has the sound-speed profile of downward refracting. In this environment, it is well known that the higher-order modes are dominantly excited by the surface source. They utilized this physical characteristic. Two hypotheses for the low-order mode subspace and the high-order model subspace were tested for the source depth discrimination. A similar study was performed by Conan et al. [10]. They defined decision metrics as the ratio of the trapped energy by low-order modes to the total energy, where the mode coefficients were extracted from the horizontal line array (HLA) data using three types of mode-filtering methods: The matched filter, least-square estimator, and regularized-least-squares estimator. In 2018, Liang et al. [11] suggested the depth discrimination method using the HLA of the acoustic vector sensors. They showed that the use of the acoustic vector sensors enhanced the accuracy of mode extraction; thus, a good performance was observed even for the short HLA. Besides the depth discrimination studies based on the mode extraction and the physical models, Du et al. [12] showed that a change of the waveguide that is invariant to the source depth in the spectrogram could be used as a clue for the depth discrimination. In an interesting study, Yang [13] demonstrated the potential of the data-based matched mode source localization with the numerical simulation. The mode parameters are completely extracted from the data; therefore, this method does not require any environmental information or assume any physical model. However, it is noted that the formulation of this method is obviously established from the physics of acoustic wave propagation. For this reason, its performance is influenced by the geometry of the receiving array.

Meanwhile, classification has been one of the key topics in the machine learning. With the prevalence of big data and the recent development of computing technology, machine learning has reemerged as an alternative for innovation in science and technology. Machine learning algorithms [16,17,18,19,20] construct a generalized model that only relies on the given data, known as “training data”. This model can be used for prediction or decision without being additionally programmed to carry out the task. Well-known machine learning algorithms include random forest (RF), support vector machine (SVM), feedforward neural network (FNN), and deep learning (DL). Specifically, the DL is known as the end-to-end machine learning algorithm that does not require preprocessing of the given data.

In this study, we explore the applicability of machine learning algorithms for the binary classification of surface and underwater sources. Four machine learning algorithms—RF, SVM, FNN and convolutional neural network (CNN)—are applied to the binary classification. Here, the CNN is a deep neural network that shows good performance in the image recognition and classification. Data for learning are modeled considering a vertical line array (VLA) by a Monte-Carlo simulation in the shallow-water environment of East Sea in South Korea. Since our study is purely data-driven, there are no concerns on the choice of a physical model or the absence of a *priori* to the ocean environment, unlike previous studies.

This paper is organized as follows. In Section 2, the normal-mode model for acoustic wave propagation is summarized and the input data format for learning is discussed. A short review of four machine learning algorithms is given in Section 3, with a focus on their hyperparameters and their optimization. Section 4 presents a performance evaluation for these four machine learning algorithms. Discussion and conclusion are given in Section 5.

## 2. Normal-Model Model and Cross-Spectral Covariance Matrix (CSDM)

**Figure 1:**
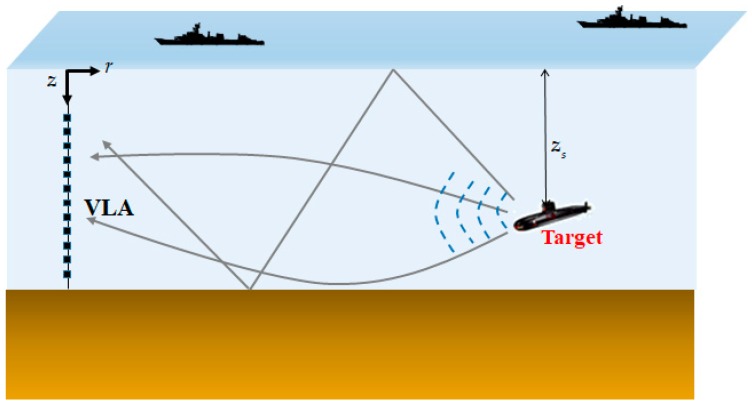
Illustration of passively receiving a target signal using the vertical line array (VLA) in the ocean environment.

For a monochromatic point source located at depth $z_{s}$ as shown in Figure 1, the normal-mode representation of the acoustic pressure field in the cylindrical oceanic waveguide [3,21] is

(1) $$p(r,z)=\frac{\sqrt{2\pi }e^{j\pi /4}}{\rho (z_{s})}\sum_{m=1}^{M}\phi_{m}(z)\phi_{m}(z_{s})\frac{e^{jk_{m}r}}{\sqrt{k_{m}r}},\;$$

where $k_{m}$ and $\phi_{m}$ are the horizontal wavenumber (normal mode) and the mode function for m^th^ mode, respectively; ρ is the water density, and M is the number of propagation modes. Using mode-amplitude $a_{m}(r,z_{s})$, Equation (1) is concisely rearranged into

(2) $$p(r,z)=\sum_{m=1}^{M}a_{m}(r,z_{s})\phi_{m}(z),\;$$

where $a_{m}(r,z_{s})=[\sqrt{2\pi }e^{j(k_{m}r+\pi /4)}/(\rho (z_{s})\sqrt{k_{m}r})]\phi_{m}(z_{s})$.

Assuming that the signal is received on an N-element VLA, Equation (2) can be written in the matrix form as

(3) $$p=\left[\begin{array}{c} p(r,z_{1})\\ \vdots \\ p(r,z_{N}) \end{array}\right]=\left[\begin{array}{ccc} \phi_{1}(z_{1}) & \ldots & \phi_{M}(z_{1})\\ \vdots & \ddots & \vdots \\ \phi_{1}(z_{N}) & \ldots & \phi_{M}(z_{N}) \end{array}\right]\left[\begin{array}{c} a_{1}\\ \vdots \\ a_{M} \end{array}\right]=\Lambda a\;.$$

Here, Λ is a rectangular N×M matrix. 

When the complex signal amplitude of the source is set as $s_{0}$, the signal model for a passive VLA including a white Gaussian noise is expressed using Equation (3) as

(4) $$p=s_{0}\;\Lambda a+n,$$

where n is the noise vector of 1×N.

By averaging over L snapshots, the phone-space cross-spectral density matrix (pCSDM) is defined as

(5) $$R_{p}=\;<pp^{H}>,\;$$

where $<\cdot >=(1/L)\sum_{l=1}^{L}{\left(\cdot \right)}_{l}$ is an ensemble average of $pp^{H}$ for each snapshot, and H indicates the conjugate transpose operator.

Meanwhile, assuming that the properties of the ocean environment are known, the VLA data can be projected into the mode-space through the inversion of Equation (4) to the mode amplitude vector. When the estimated mode amplitude vector is represented by $\tilde{a}$, the mode-space cross-spectral density matrix (mCSDM) can be defined as

(6) $$R_{m}=\;<\tilde{a}{\tilde{a}}^{H}>.\;$$

The pCSDM of Equation (5) is built with the pressure signals received at the VLA. It is difficult to see any features of the surface and underwater source directly from the pCSDM. However, as mentioned in the introduction, it is well-known that the mCSDM shows a remarkable feature for the source depth in a shallow-water environment, especially for a downward-refracting sound speed profile. This is because the lower-order modes are excited for the underwater sources, whereas the higher-order modes are excited for both sources. Therefore, the projection into the mode space can be regarded as a feature extraction of input data.

In this study, we use the pCSDM and mCSDM as an input data. Since the CSDM is conjugate symmetric, the diagonal and upper triangular part is chosen as input data for the machine learning algorithms of RF, SVM, and FNN, and is vectorized. Moreover, we consider two cases of the absolute value of CSDM, and the real and imaginary values of CSDM. Thus, when the size of CSDM is k×k, the number of input samples for the absolute value and the real and imaginary values is k(k+1)/2 and k(k+1). In total, these four types of input data are fed to three machine learning algorithms, respectively. For the CNN [22,23,24], the matrix format is used as input. The two-dimensional absolute matrix of CSDM and the three-dimensional matrix that consists of the real and imaginary matrix of CSDM are respectively chosen as an input in the CNN, and their sizes are k×k and k×k×2.

## 3. Machine Learning Algorithms

In this section, we describe machine learning algorithms used in the study. The classification problem is ideally defined by the equation

(7) $$t_{k}=H(x),$$

where x is the input data in P dimensions, $t_{k}$ represents the k^th^ class label to which the input data x belongs to, and H is a nonlinear system function mapping x onto $t_{k}$. In many realistic problems, system function H is unknown, or hard to model analytically. Machine learning algorithms try to overcome this difficulty by building the system function from the training set. Because several good references [16,17,18,25] for machine learning exist, we concisely review the theory of RF, SVM, FNN, and CNN and their hyperparameter tuning.

### 3.1. Random Forest

The RF is a nonlinear classifier that consists of many decision trees uncorrelated to one another. A decision tree is a decision tool that recursively partitions the input data into a number of regions using the greedy approach. For each decision tree, the cutoff thresholds used to partition the entire region are determined by learning subsets of training data, selected uniformly from the training data set. The RF algorithm in the learning part is listed in Algorithm 1.

**Algorithm 1.** RF learning algorithm: Learning algorithm of random forest.
**for**
m=1:M

(a)Draw N samples from the training data set.(b)Grow a decision tree $f_{m}(\cdot )$ using the sampled data, by recursively repeating the following steps for each terminal node of the tree until the tree has the maximum size.(i)Select D variables randomly from P variables.(ii)Choose the best attribute/split-point among the D
(iii)Split the node into two sub-branches
**end**


Given input data $x_{i}$, assume that m^th^ decision tree model is set to be $t_{k,i}=f_{m}(x_{i})$. For RF classification, output $t_{k,i}$ represents the k^th^ class label of $x_{i}$. Next, the RF model is defined as below:

(8) $$t_{k,i}=f(x_{i})=\underset{t_{k,i}}{\arg\max}\sum_{m=1}^{M}I[f_{m}(x_{i}),{\hat{t}}_{k,i}]$$

where M is the number of decision trees, and

(9) $$I[f_{m}(x_{i}),{\hat{t}}_{k,i}]=\{\begin{array}{l} 1,f_{m}(x_{i})={\hat{t}}_{k,i}\;\\ 0,\;\mathrm{otherwise} \end{array}.$$

Since the RF model is an ensemble mode of M estimates, which is called bagging (bootstrap aggregating), the variance of an estimate can be reduced. For the use of RF model, it is important to determine the number of decision trees M, which is a hyper parameter. If M is too large, the enhancement of the performance is stagnant, whereas the computational complexity is increasing. If M is too small, the accuracy of an estimate will be poor. In this study, we change the number of decision trees in the range of $\left[2^{1},2^{2},\cdots ,2^{11}\right]$ and choose the value that produces the best performance.

### 3.2. Support Vector Machine

**Figure 2:**
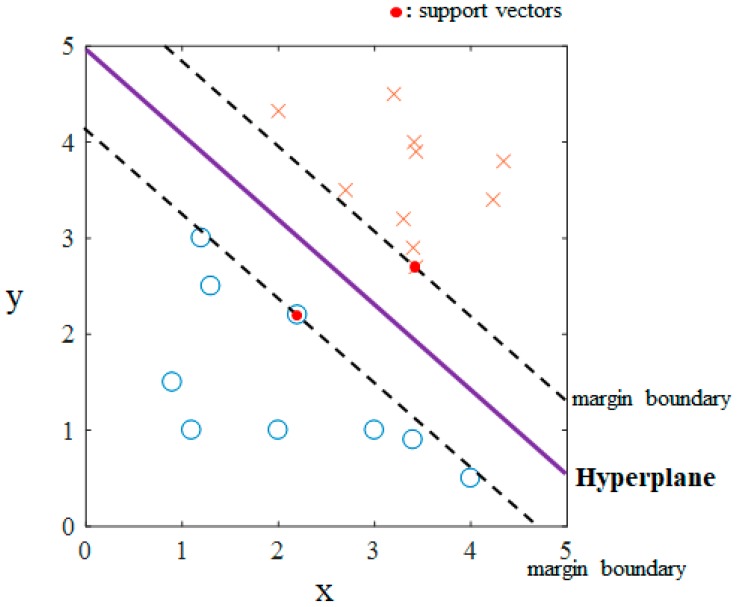
Example of linear support vector machine binary classification in two dimensions. The symbols ‘O’ and ‘X’ represent the data in each class.

The SVM classifies the data geometrically using a decision boundary, called the hyperplane, as shown in Figure 2. The decision boundary is determined in the process of learning by maximizing the margin distance from the hyperplane to the support vectors. For a nonlinear binary classification, the SVM model is formulated as

(10) $$y_{i}=f(x_{i})=w^{T}\phi (x_{i})+b,\;$$

where $y_{i}$ is the pseudo-output of the SVM model, $w^{T}$ and b are the unknown weight vector and bias, found by learning the data, and ϕ(⋅) is a nonlinear mapping function. If $y_{i}>0$ and if $y_{i}<0$ on the hyperplane of $w^{T}\phi (x_{i})+b=0$, $x_{i}$ is labeled as $t_{1,i}=1$ and $t_{-1,i}=-1$, respectively.

Given N samples of training data, the optimizing problem for (N+1) unknown parameters of Equation (10) is formally described by

(11) $$\underset{w,b}{\arg\min}\;\frac{1}{2}{\left\|w\right\|}^{2}\;subject\;to\;t_{k,i}[w^{T}\phi (x_{i})+b]\geq 1,\;n\;=\;1,\ldots ,N.$$

The constraints of Equation (11) can be more relaxed by introducing slack variables $\xi_{n}$ thus allowing for errors as below.

(12) $$\underset{w,b,\xi_{n}}{\arg\min}\;\frac{1}{2}{\left\|w\right\|}^{2}+C\sum_{n=1}^{N}\xi_{n}\;subject\;to\;t_{k,i}[w^{T}\phi (x_{i})+b]\geq 1-\xi_{n},\;\xi_{n}\geq 0,\;n\;=\;1,\ldots ,N.$$

where C is a regularization parameter that controls the overfitting; C > 0.

The dual form of Equation (12) can be derived using the Lagrangian form as

(13) $$L(\alpha_{1},\cdots ,\alpha_{N})=\sum_{i=1}^{N}\alpha_{i}-\frac{1}{2}\sum_{i=1}^{N}\sum_{j=1}^{N}\alpha_{i}\alpha_{j}t_{k,i}t_{k,j}K(x_{i},x_{j})\;subject\;to\;\sum_{i=1}^{N}a_{i}t_{k,i}=0,0\leq \alpha_{i}\leq C,\;n\;=\;1,\ldots ,N.$$

where $K(x_{i},x_{j})=\phi^{T}(x_{i})\cdot \phi (x_{j})$ is the kernel function acting as a similarity measure between data sets. There are several types of kernel functions. Among them, we use the Gaussian radial basis function (RBF) kernel,

(14) $$K(x_{i},x_{j})=e^{-\gamma {\left\|x_{i}-x_{j}\right\|}_{2}^{2}},\;$$

where γ is a parameter that sets the dispersion of the kernel and has a non-negative value. Note that the feature space of the Gaussian RBF is infinite-dimensional.

Regularization parameter C and parameter of Gaussian RBF γ are tuned as hyper-parameters. A high C may lead to overfitting, whereas a low C possibly maximizes the margin that leads to the underfitting. The value of γ defines how far the influence of a single training sample reaches. For a high γ, the SVM model can overfit and give low bias and high variance. A low γ is closely related to underfitting, which represents the trend with a high bias and low variance. We optimize these two hyperparameters of C and γ in the range from $10^{-3}$ to $10^{2}$ and from $10^{-4}$ to $10^{1}$, respectively.

### 3.3. Feedforward Neural Network

**Figure 3:**
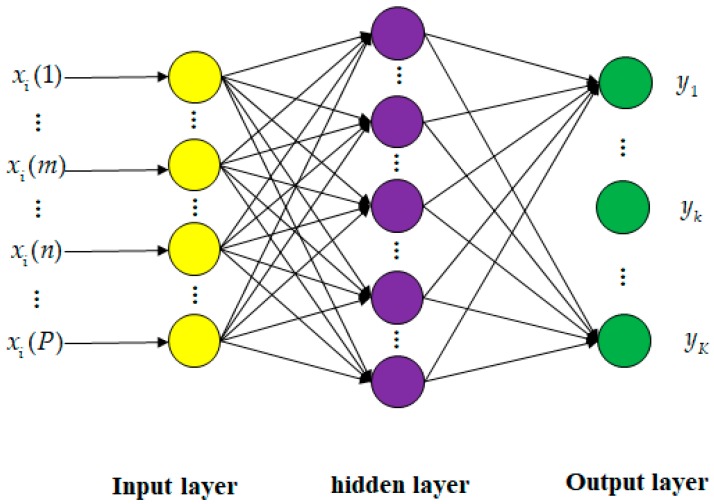
Structure of feedforward neural network with one hidden layer for classification with k classes.

A feedforward neural network is a multi-layered network of perceptrons. A perceptron represents an artificial and simplified model of a biological neuron, acting as a unit classifier. The FNN consists of an input layer, hidden layers, and an output layer. Figure 3 shows a typical example of the FNN with one hidden layer and the output layer with binary outputs. Every perceptron in a layer is connected to perceptrons on the next layer with the weights. The output of a perceptron, called activations, is transformed using a nonlinear activation function. This activation function makes the FNN a nonlinear classifier in deep sense.

For example, the FNN model in Figure 3 is mathematically formulated as

(15) $$y_{k}=f(x_{i})=\sigma_{0}\left(\sum_{m=1}^{M}w_{km}^{\left(2\right)}\sigma \left(\sum_{n=1}^{N}w_{mn}^{\left(1\right)}x_{i,n}+w_{m0}^{\left(1\right)}\right)+w_{k0}^{\left(2\right)}\right),k=1,2$$

where $w_{km}^{\left(2\right)}$ and $w_{mn}^{\left(1\right)}$ are unknown weights, $w_{k0}^{\left(2\right)}$ and $w_{m0}^{\left(1\right)}$ are biases, and $y_{k}$ is the pseudo-output of the k^th^ perceptron in the output layer. Here, σ(x) is an activation function, for which we use the rectified linear unit (ReLU) function,

(16) σ(x)=max(x,0).

Specifically, $\sigma_{0}$ is an activation function in the output layer. For the classification, the softmax function is commonly used as an activation function:

(17) $$y_{k}=\sigma_{0}(a_{k})=\frac{\exp(a_{k})}{\sum_{m=1}^{M}\exp(a_{m})},$$

where M is the number of outputs in the output layer. As shown in Equation (16), the output of the softmax function is between 0 and 1, and the summation of outputs in the output layer has to be 1. This property is very useful because the value of the pseudo-output can be treated as a probability.

In the FNN, the unknown weights and biases are determined by solving the optimization problem for the loss function. For the classification, cross-entropy error function $E_{i}$ for given data sample $x_{i}$ is widely used as a loss function:

(18) $$E_{i}(w_{mn}^{\left(l\right)})=-\sum_{k=1}^{K}t_{k,i}\ln y_{k}(x_{i}),\;$$

where $t_{k,i}$ is the k^th^ class label for give data $x_{i}$, categorized by one-hot encoding. For N training samples, loss function $E(w_{mn}^{\left(l\right)})$ is generalized as the arithmetic mean of the cross-entropy error functions from i=1 to i=N, $E(w_{mn}^{\left(l\right)})=(1/N)\sum_{i=1}^{N}E_{i}$. The corresponding optimization problem is formulated as $\underset{w_{mn}^{\left(l\right)}}{\arg\min}\;(1/N)\sum_{i=1}^{N}E_{i}$, and usually solved by the gradient descent method. Practically, the deterministic gradient descent method using full training data sets is not preferred due to its computational inefficiency. A so-called minibatch gradient descent method is more popular and its outline is listed in Algorithm 2.

A neural network can better infer hidden relationships from complex nonlinear data than other machine learning algorithms. However, determining the proper neural network architecture can be a major drawback. To design a better neural network, many hyperparameters such as the number of layers, number of neurons, depth of network, width of network, type of activation function, and several hyperparameters for the optimizer are repeatedly adjusted.

**Algorithm 2**. Minibatch gradient descent algorithm: Algorithm of minibatch stochastic gradient descent method.Initialize unknown parameters.
**for**
i=1:N

(a)Sample a minibatch of size m from the training data.(b)Compute the gradient estimate using all data of the minibatch.(c)Update the parameters.
**end**


In this study, we built a deep FNN with 4 hidden layers and 1024 layer width (neurons) to explore the potential of FNN. The Nesterov momentum stochastic gradient descent (SGD) method with minibatch is chosen with the momentum parameter of 0.9. The size of minibatch is 64, and the learning rate and epoch are determined from the validation test. In addition, the dropout technique is used to reduce the overfitting in the deep neural network. The dropout is a technique to drop out some neurons chosen randomly during the learning. The dropout probability is set to be 0.5.

### 3.4. Convolutional Neural Network

Unlike the FNN, the convolutional neural network learns from images, which are basically three-dimensional (row, column, and pixel). Thus, we do not need to vectorize the input image into one-dimensional data for the CNN. In this way, the CNN shows the best performance in image recognition. 

The hidden layer of the CNN typically consists of a convolutional layer, rectified linear unit (ReLU) layer, and pooling layer. In the convolutional layer, the convolution of the input feature map with the filter matrix is performed. This operation helps to extract any feature from the local region of image, and decreases the burdens of enormous unknown weights, compared with the fully connected neural network. Of course, the components of the filter matrix, stride size, and padding size are properly determined. The pooling layer reduces the size of the feature map by clustering the local region at the present layer and mapping one neuron at the next layer. The mapping is carried out by max pooling or average pooling. In the hidden layer close to the output layer, the affine layer (or fully connected layer) is frequently used in the typical CNN architectures.

**Figure 4:**
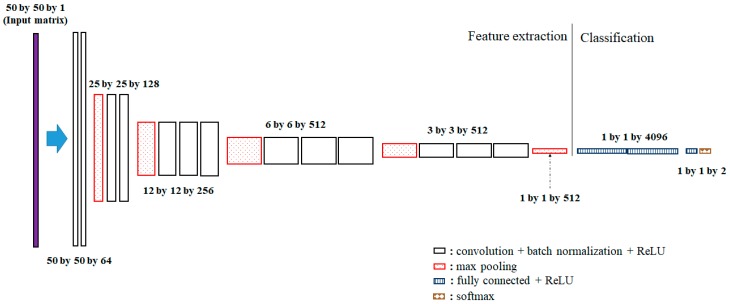
VGG16 architecture and its dimensions used in this study. Note that the input data has a matrix form and the fully connected layers are used for classification.

In this study, we use the architecture of VGG16 proposed by Simonyan and Zisserman [25] as shown in Figure 4, rather than building a new CNN architecture. The VGG16 is a deep neural network that contains 13 convolutional layers and 3 affine layers. Additionally, the batch-normalization layer [26] to prevent the distribution of the features from varying at every step is inserted between the convolutional layer and the activation layer. Additionally, the dropout technique is applied between the affine layers. In VGG16, the components of the filter matrix are initialized by He initialization [27].

## 4. Results

### 4.1. Data Modeling Considering a VLA

**Figure 5:**
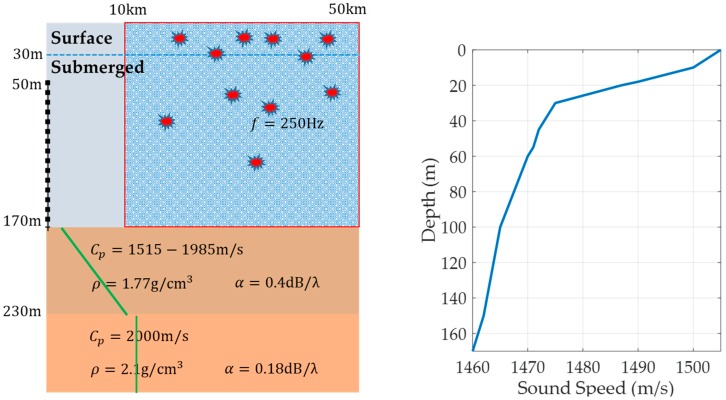
Shallow water environment of MAPLE4 experimental site (**right** figure). Red symbols represent random positions of targets in the ocean. The **left** figure shows an ocean environment with a vertical line array. The right figure is the sound speed profile.

Acoustic data are numerically simulated with the normal-mode propagation model, KRAKEN [21,28], developed by Porter in the MAPLE4 ocean environment of the East Sea of South Korea, as shown in Figure 5. This site is typically a shallow-water environment with the downward refracting sound speed profile. The ocean bottom consists of the sediment layer with varying properties and the rock basin. The sound speed profile is measured with the expendable bathythermograph, and the bottom properties are inverted from geological and acoustical in-situ measurements. The VLA has 48 elements and spans the lower 70% of the water column. The interval between acoustic sensor is 2.5 m.

We assume that the monochromatic target with the frequency 250 Hz is located at a position within the region with the depth of 0 m to 170 m and the range of 10 km and 50 km, and the source position is uniformly distributed. Additionally, the signal-to-noise ratio (SNR) is assumed to be uniformly distributed between 2 dB and 5 dB. By a Monte-Carlo simulation, 6010 realizations with different source ranges and different SNRs are uniformly generated, called TEST1 data in this study. To avoid the class imbalance problem in machine learning, the frequency count of the surface target and underwater target is set to be equally likely: Samples in each depth region are generated uniformly. We use the depth of 30 m as the reference depth.

Apart from the TEST1 data, 965 other data samples, called TEST2 data, are generated to evaluate the robustness of the machine learning algorithms. The TEST2 data are collected under the same conditions as the TEST1 data, except that the SNR is uniformly chosen between −1 dB and 0 dB. Thus, the quality of TEST2 data is poorer than of the TEST1 data. As well, the simulation is performed on a PC with Intel i-7 CPU and NVIDIA GTX1080 GPU. For one test data set, all machine learning classifier trained have provided the results in real time (<0.5 s).

### 4.2. Input Data and Mode Signal Processing

For the TEST1 data, the preprocessing of the input data is performed according to the following procedure:

1. Using L snapshots, the pCSDM and mCSDM are obtained using Equations (5) and (6), respectively. In the mCSDM, the mode amplitudes are estimated using the pseudo-inverse filter given as $H={\left(\Lambda^{H}\Lambda \right)}^{-1}\Lambda^{H}$;
2. As described in the introduction, each CSDM data sample is vectorized and converted into two types of format: Absolute value data and complex value data. In total, four types of data format are used. Note that the CNN does not need vectorization;
3. All data samples are labeled for two hypotheses ($H_{0}:z_{s}\leq z_{ref},H_{1}:z_{s}>z_{ref}$ ). Here, $z_{ref}$ is the reference depth for discriminating the surface/underwater targets;
4. The vectorized pCSDM and mCSDM data samples are divided into the training set, validation set, and test set. Their sizes are 4058, 1078, and 874, respectively. The validation set is used to adjust the hyperparameters of machine learning algorithms. The training set is used to train the machine learning algorithm. The performance of the algorithm is only evaluated on the test set. The hyperparameters used in four machine learning algorithms are listed in Table 1.

TEST2 data are preprocessed in steps 1, 2, and 3.

### 4.3. Principal Component Analysis

**Figure 6:**
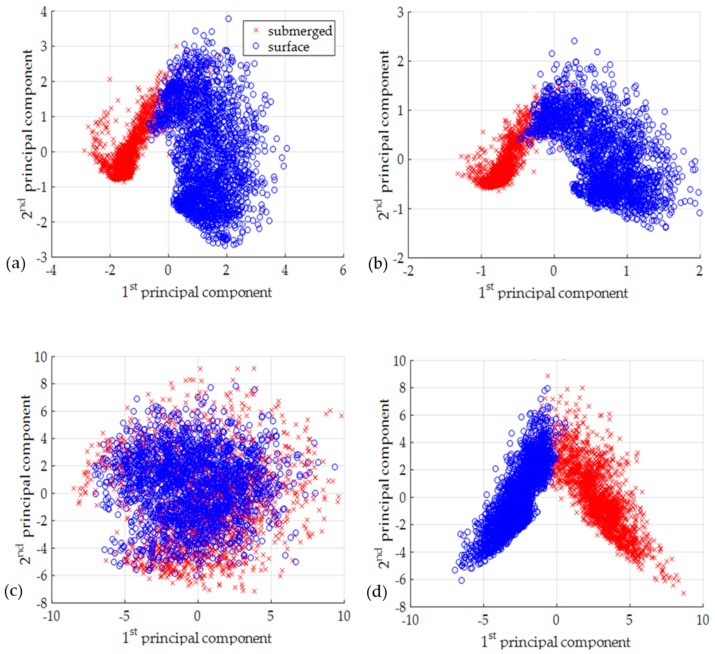
Scattered plots using the first and second principal components extracted from each input data format, which is (**a**) the absolute mCSDM, (**b**) the complex mCSDM, (**c**) the absolute pCSDM, and (**d**) the complex pCSDM.

To check the separability of four data formats (absolute pCSDM, complex pCSDM, absolute mCSDM, and complex mCSDM) in advance, the principal component analysis (PCA) is applied. The PCA can be done with the singular value decomposition of the data matrix after mean normalization. We extract the first and second principal components from labeled data, and visualize them in two-dimensional domain, as shown in Figure 6. The absolute pCSDM data visually fails to separate different clusters, whereas clusters look more separated with other three data formats. It is interesting that the absolute mCSDM data as well as the complex mCSDM data also exhibits the unique features for two classes. In Equation (2), the amplitude term of $a_{m}(r,z_{s})$ is clearly shown to be a function of source depth. This is the reason that the absolute mCSDM data are separable in the PCA.

### 4.4. Classification Results

From the TEST1 data, we train RF, SVM, FNN, and CNN binary classifiers. The performance of the algorithm is assessed by the percent error:

(19) $$E_{p}=\frac{n_{f}}{N}\times 100\;(\%),\;$$

where N is the total number of test samples, and $n_{f}$ is the number of misclassified data among them.

The percent errors for machine learning algorithms are listed in Table 2. For the test set of TEST1 data, which is expected to have the same distribution as the training set, the absolute mCSDM data shows better performance than the complex mCSDM data for all cases except the RF using TEST1 data and the SVM using TEST2 data, but the difference between them is tiny and below 0.35%. For the mCSDM data, the FNN and CNN are the best with the TEST2 data and the TEST1 data, respectively.

However, the pCSDM data displays a different trend with the mCSDM data. The absolute pCSDM data gives the worst results in all cases, with a percent error that is much higher than that of other data formats. This observation can also be supported with the results of PCA analysis in Figure 6. In Table 2, an interesting point is that the complex pCSDM data gives good performance comparable to two mCSDM data formats and is even the best in the CNN. Considering that the pCSDM data does not require any preprocessing using the parameters of the ocean environment, the use of the pCSDM data has a big advantage. The SVM is ranked top for the absolute pCSDM data. We are not exactly sure how this works. For eight cases (4 data formats, 2 datasets), the CNN is top-ranked in four data inputs. The next ranking is the FNN and SVM, which tie. However, when the absolute pCSDM case is excluded, the FNN is superior. 

To extract more informative results, we eliminate the results using the absolute pCSDM data, biased from others, and calculate the arithmetic mean and the range (largest data minus smallest data) of the percent errors for each machine learning algorithm. The mean percent errors are 3.17, 2.02, 1.88, and 1.65 for RF, SVM, FNN, and CNN, respectively. The ranges of the percent errors are 2.8, 1.0, 0.68, and 1.57. Judging by the mean percent error, the CNN remains the best. However, the CNN seems to have a moderate variability for input data cases. For further analysis, we calculate the coefficient of variation (CV) defined as the ratio of the standard deviation to the mean, which shows the relative variability. The arithmetic mean and range are used for the estimations of the true standard deviation and mean. The results are 0.88, 0.49, 0.36, and 0.95. The FNN has lowest relative variability for data formats, and the CNN is the opposite. To evaluate the robustness of the machine learning system to the noisy data, the ratio between the percent errors is listed in Table 3. In Table 3, the percent errors of all learning algorithm increase, because the TEST2 data was acquired with the different SNR. Specifically, the ratio of the CNN is at approximately 2.0, except for the case of the complex pCSDM data. It means that the complex pCSDM data format may be suitable for the prediction using the CNN in the noisy environment. The RF with the complex mCSDM data is worst for the classification of the TEST2 data. However, the overall increase of percent error is not large for all machine leaning algorithms.

**Figure 7:**
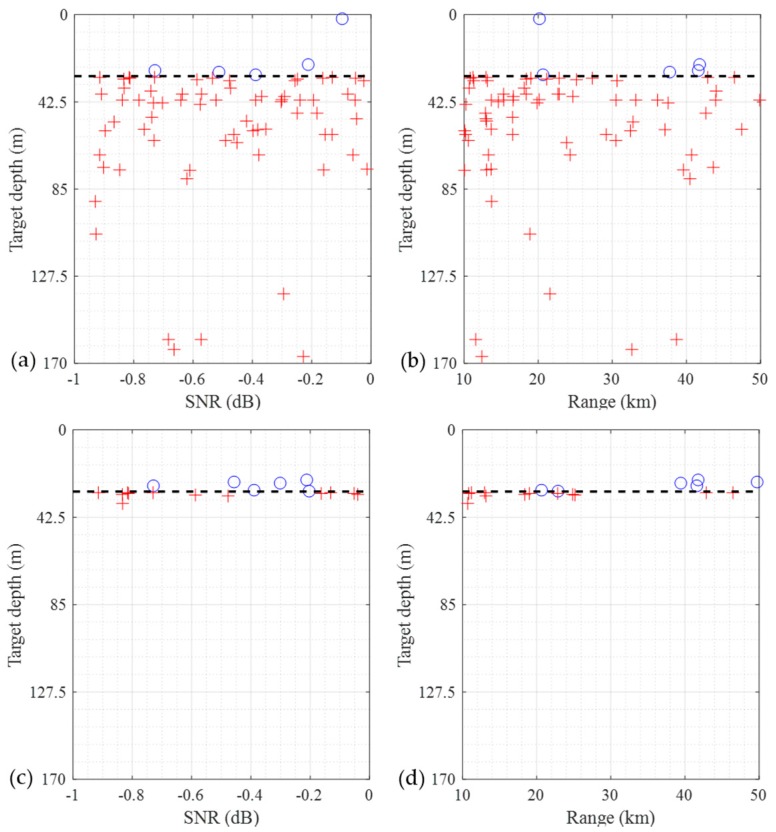
Scatter plots for the CNN misclassification using the absolute pCSDM (Figure 7a,b) and complex PCSDM (Figure 7c,d) in the TEST2 data as a function of the SNR or the range. The symbols ‘O’ and ‘+’ represent the misclassified positions of the underwater targets and surface targets, respectively.

Finally, a scatter-plot of the CNN misclassification from the TEST2 data is shown in Figure 7. The absolute pCSDM data and the complex pCSDM data is used as an input data. Figure 7a,b show the results using the absolute pCSDM data. The symbol ‘O’ represents the locations of underwater targets misclassified as surface targets. The symbol ‘X’ does the opposite. As shown in Figure 7a,b, underwater targets are misclassified more often than surface targets. This is because the same number of data is sampled for each region, although the underwater region is much broader. Similar trend is observed in Figure 7c,d, where the complex pCSDM data is used. However, the misclassification of the underwater targets is significantly reduced due to the superiority of the data format. As seen, all misclassifications occur near the reference depth. 

## 5. Discussion and Conclusions

In this paper, we apply four machine learning algorithms—RF, SVM, FNN, and CNN—to the underwater/surface target discrimination problem in a shallow-water waveguide using the vertical line array. Four datasets—the complex pCSDM, absolute pCSDM, complex mCSDM, and absolute mCSDM—are used as input data. To the first and second datasets, no preprocessing is applied except for the ensemble average of CSDM. However, the remaining two datasets are preprocessed by the mode-filtering, which should use the information of ocean environment. The PCA analysis shows that the complex pCSDM, complex mCSDM, and absolute mCSDM have good discriminative features, while the features of the absolute pCSDM are invisible in the PCA analysis. It indicates that the phase information in the phone-space data is necessary for depth discrimination, but it is not true for the mode-space data. This is because the source depth information in the mode representation is explicitly included in the mode function $\phi_{m}(z_{s})$. 

We reach the following five conclusions based on the classification results. First, the machine learning algorithms show good performance for underwater/surface target discrimination problem. For all algorithms, the percent error is less than 5%. Second, the CNN shows the best performance in the measure of low percent error but shows the highest relative variability in the measure of CV. Third, we confirm that all machine learning models work well in different environments with lower SNR. This may be the inherent ability of the machine learning or the combined effect with the ensemble average of the CSDM. For the generalization issues of machine learning, more research will be needed in the future. Fourth, the machine learning using the complex pCSDM gives good results comparable with that using the complex mCSDM and does not require a *priori* information for the ocean environment. This finding supports the usefulness of the phone-spaced data in machine learning. Finally, we conclude that the FNN with deep layers shows good performance comparable with the CNN for several measures. In particular, the FNN is better in the measure of prediction listed in Table 3.

## Figures and Tables

**Table 1:** Hyperparameters used in four machine learning algorithms.

	pCSDM (Abs.)	pCSDM (Real+Imag.)	mCSDM (Abs.)	mCSDM (Real+Imag.)
**RF**				
Number of trees	256	256	64	32
Max feature	log2	log2	sqrt	log2
**SVM**				
C	10	10	100	100
γ	0.01	0.1	0.1	0.1
**FNN**				
Number of hidden layers	3	3	3	3
Learning rate	0.001	0.001	0.001	0.001
**CNN**				
Learning rate	0.0001	0.0001	0.0001	0.0001

**Table 2:** Classification results (percent error). The bold font indicates the minimum percent error among four machine learning algorithms for each dataset. The underscore shows the best result for each algorithm.

	pCSDM (Abs.)	pCSDM (Real+Imag.)	mCSDM (Abs.)	mCSDM (Real+Imag.)
**RF**				
TEST1(test set)	7.09	2.79	2.52	2.17
TEST2	12.12	2.85	3.73	4.97
**SVM**				
TEST1(test set)	**3.20**	1.72	1.49	1.83
TEST2	**5.49**	2.18	2.49	2.38
**FNN**				
TEST1(test set)	4.81	1.95	1.60	1.72
TEST2	7.56	2.28	**1.76**	**1.97**
**CNN**				
TEST1(test set)	3.43	**1.49**	**0.92**	**1.14**
TEST2	7.67	**1.87**	1.97	2.49

**Table 3:** Ratio between the percent errors in the TEST2 data and the TEST1 data (test set). The bold font indicates the minimum ratio among four machine learning algorithms for each dataset. The underscore shows the best result for each algorithm.

	pCSDM (Abs.)	pCSDM (Real+Imag.)	mCSDM (Abs.)	mCSDM (Real+Imag.)
RF	1.71	**1.02**	1.48	2.29
SVM	1.72	1.27	1.67	1.30
FNN	**1.57**	1.17	**1.10**	**1.15**
CNN	2.24	1.26	2.14	2.18

## References

[1] Premus V. (1999). Modal scintillation index: A physics-based statistic for acoustic source depth discrimination. J. Acoust. Soc. Am..

[2] Nicolas B., Mars J.I., Lacoume J. (2006). Source depth estimation using a horizontal array by matched-mode processing in the frequency-wavenumber domain. EURASIP J. Appl. Signal Process..

[3] Kim K., Seong W., Lee K. (2010). Adaptive surface interference suppression for matched-mode source localization. IEEE J. Oceanic. Eng..

[4] Tolstoy A. (1992). Matched Field Processing for Underwater Acoustics.

[5] Steinberg B.Z., Beran M.J., Chin S.H., Howard J.H. (1991). A neural network approach to source localization. J. Acoust. Soc. Am..

[6] Ozard J.M., Zakarauskas P., Ko P. (1991). An artificial neural network for range and depth discrimination in matched field processing. J. Acoust. Soc. Am..

[7] Too G.J., Lin E., Hsieh Y. Source localization based on ray theory and artificial neural network. Proceedings of the OCEANS.

[8] Yang T.C. (1987). A method of range and depth estimation by modal decomposition. J. Acoust. Soc. Am..

[9] Premus V.E., Backman D. A matched subspace approach to depth discrimination in a shallow water waveguide. Proceedings of the Forty-first Asilomar Conference on Signals, Systems & Computers.

[10] Conan E., Bonnel J., Nicolas B., Chonavel T. (2017). Using the trapped energy ratio for source depth discrimination with a horizontal line array: Theory and experimental results. J. Acoust. Soc. Am..

[11] Liang G., Zhang Y., Zhang G., Feng J., Zheng C. (2018). Depth discrimination for low-frequency sources using a horizontal line array of acoustic vector sensors based on mode extraction. Sensors.

[12] Du J., Zheng Y., Wang Z., Cui H., Liu Z. Passive acoustic source depth discrimination with two hydrophones in shallow water. Proceedings of the OCEANS.

[13] Yang T.C. (2014). Data-based matched-mode source localization for a moving source. J. Acoust. Soc. Am..

[14] Premus V., Helfrick M.N. (2013). Use of mode subspace projections for depth discrimination with a horizontal line array. J. Acoust. Soc. Am..

[15] Conan E., Bonnel J., Chonavel T., Nicolas B. (2016). Source depth discrimination with a vertical line array. J. Acoust. Soc. Am..

[16] Hastie T., Tibshirani R., Friedman J. (2001). The Elements of Statistical Learning: Data Mining, Inference, and Prediction.

[17] Goodfellow I., Bengio Y., Courville A. (2016). Deep Learning.

[18] Niu H., Reeves E., Gerstoft P. (2017). Source localization in an ocean waveguide using supervised machine learning. J. Acoust. Soc. Am..

[19] Russell S., Norvig P. (2010). Artificial Intelligence: A Modern Approach.

[20] Ordonez F.J., Roggen D. (2016). Deep convolutional and LSTM recurrent neural networks for multimodal wearable activity recognition. Sensors.

[21] Jenson F.B., Kuperman W.A., Porter M.B., Schmidt H. (2011). Computational Ocean Acoustics.

[22] Xu K. (2017). North Atlantic right whale call detection with very deep convolutional neural networks. J. Acoust. Soc. Am..

[23] Hirvonen T. Classification of spatial audio location and content using convolutional neural networks. Proceedings of the AES 138th Convention.

[24] Ferguson E.L., Ramakrishnan R., Williams S.B., Jin C.T. Convolutional neural networks for passive monitoring of a shallow water environment using a single sensor. Proceedings of the ICASSP.

[25] Simonyan K., Zisserman A. Very deep convolutional networks for large-scale image recognition. Proceedings of the ICLR.

[26] Loffe S., Szegedy C. (2015). Batch normalization: Accelerating deep network training by reducing internal covariate shift. arXiv.

[27] He K., Zhang X., Ren S., Sun J. (2015). Delving deep into rectifiers: Surpassing human-level performance on ImageNet classification. arXiv.

[28] Ocean Library. http://oalib.hlsresearch.com/Modes.

